# Editorial: Cutaneous vasculitis and vasculopathy

**DOI:** 10.3389/fmed.2025.1557558

**Published:** 2025-02-12

**Authors:** Erkan Alpsoy, Cord H. Sunderkötter, Warren Piette

**Affiliations:** ^1^Department of Dermatology and Venereology, School of Medicine, Akdeniz University, Antalya, Türkiye; ^2^Department of Dermatology and Venereology, University Hospital Halle (Saale), Martin-Luther-University Halle-Wittenberg, Wittemberg, Germany; ^3^Division of Dermatology, John H Stroger Hospital of Cook County, Chicago, IL, United States; ^4^Department of Dermatology, Rush University Medical Center, Chicago, IL, United States

**Keywords:** vasculitis, small vessel vasculitis (SVV), cutaneous vasculitis, vasculopathia, IgA vasculitis

Vasculitis is a disorder group with inflammation and necrosis of blood vessel walls, causing hemorrhagic and ischemic features. It can appear in any organ of the body, and can influence blood vessels of any size. The severity of vasculitis can range from mild and temporary to life-threatening. The skin is commonly affected by vasculitis, with small-vessel vasculitis being the most prevalent form. Cutaneous vasculitis can occur as part of systemic vasculitis, either as a skin-limited or skin-dominant expression, or as a variant of the systemic condition. Eventually, it may be an isolated-vessel inflammation of the skin. The term (occluding) vasculopathy is used to describe the blockage of blood flow in a vessel due to occludung events such as emboli, thrombi, cryoproteins, high blood visosity or proliferative processes of the vessel wall (while livedoid vasculopathy is a term for a special entity withing this group). Vasculopathy is sometimes also used as a broad term to encompass any disorder affecting the blood vessels.

Dermatologists have an advantage in recognizing and diagnosing cutaneous vasculitis early. This is because vasculitis often involves the skin, which is visible and easily accessible for examination and biopsy. Additionally, the presence and/or spectrum of skin lesions can indicate severe systemic vasculitis. This special “Dermatology” Research Topic is dedicated to focusing on the dermatological aspect of the disease. Taking into account the multisystemic nature of the disease, we also tried to deal with cutaneous vasculitis and vasculopathy in all respects. Therefore, this title is expected to be of interest to a wide range of disciplines. Our focus is on the current knowledge of epidemiology, etiopathogenesis, clinical features, diagnosis, differential diagnosis and therapeutic approaches for the treatment of cutaneous vasculitis and vasculopathy.

Cutaneous vasculitis includes various conditions, ranging from limited skin involvement to severe systemic forms. In the last years interdiscplinary agreement has been reached on the terminology for cutaneous vasculitides. In this special supplement, we analyze the latest advancements and open questions in the terminology of cutaneous vasculitis. Although the skin is frequently affected by vasculitides, it was not until 2018 that a specific set of terms, based on the Chapel Hill Consensus Conference (CHCC) nomenclature, was introduced to identify the distinct features of cutaneous vasculitides. Caproni et al.'s article emphasizes the importance of the Dermatologic Addendum to CHCC2012 (D-CHCC) and its impact on the scientific community, as discussed in “The impact on the scientific community of the 2018 addendum to the CHCC (Chapel Hill Consensus Conference).”

Immune complex vasculitides present with inflammation of the vessel walls associated with perivascular deposition of immunoglobulins, particularly immune complexes. This group includes systemic and skin-restricted IgA vasculitis variants, cryoglobulinemic vasculitis, rheumatoid, lupus and hypocomplementaemic vasculitides, serum sickness as well as cutaneous IgM/IgG-vasculitis or recurrent macular vasculitis (such as hypergammglobulinemic or exercise-induced). Sunderkötter et al. provide a comprehensive overview of the pathophysiology and clinical manifestations of immune complex vasculitides, revealing that some pathomechanisms, e.g. in IgA vasculitis, may differ considerably from the mere concept of serum sickness or the Arthus reaction.

In the “*Recent topics related to etiology and clinical manifestations of cutaneous arteritis*” title, Ikeda underlines that adenosine deaminase 2 deficiency cases are included among the cases diagnosed with cutaneous arteritis. Due to clinical similarities with cutaneous arteritis but differences in treatment approaches, if cutaneous arteritis is diagnosed or developed, especially in early childhood, it should prompt consideration of ADA 2 deficiency as a possible cause.

In two separate reviews, we tried to analyze the most recent literature on the clinical and immunohistopathological features of cutaneous vasculitis caused by systemic SARS-CoV-2 infection and cutaneous vasculitis secondary to SARS-CoV-2 vaccine. While Corrà et al. specifically focus on possible underlying pathogenetic mechanisms, Maronese et al. perform a detailed clinicopathological evaluation.

Livedoid vasculopathy is a chronic, relapsing, thrombo-embolic disease characterized by occlusion of the dermal vessels of the lower extremities. Burg et al. provide an overview of the current literature on livedoid vasculopathy, provide a diagnostic and therapeutic approach, and review diseases that fall under the differential diagnosis of livedoid vasculopathy. In their comprehensive review, Seguí and Llamas-Velasco also provide a detailed analysis of the pathogenesis, associations, clinical features, and treatment strategies associated with livedoid vasculopathy.

Kim et al. focus on the pathogenesis of vasculitis in Behçet's disease, which is classified as variable vessel vasculitis. The authors provide updated clinical information and therapeutic recommendations for mucocutaneous Behçet's disease, with a special emphasis on idiopathic immune-mediated vasculitis.

In the review titled “Cutaneous vasculitis; An algorithmic approach to diagnosis,” Alpsoy presents a systematic diagnostic approach. The approach combines current literature knowledge and the author's expertise in the field to offer a rational framework for selecting the most suitable diagnostic methods.

Micheletti discusses the treatment of cutaneous vasculitis, emphasizing that the choice of treatment depends on the type, severity, and patient comorbidities. Well-planned treatment can achieve disease remission with minimal drug toxicity. The treatment of systemic vasculitis is evolving toward more targeted therapies based on improved understanding of the disease.

In conclusion, we have endeavored to elucidate the multifaceted aspects of cutaneous vasculitis and vasculopathy in a comprehensive manner, utilizing the expertise of renowned figures in this field. In this Research Topic, the aim has been to write each title in an up-to-date and concise manner. Furthermore, evidence-based algorithmic approaches have been proposed in appropriate topics. It is our hope that this Research Topic will prove a valuable resource for physicians engaged in the clinical management of cutaneous vasculitis and vasculopathy.

